# Editorial: Single cell analysis of bacteria-host interaction

**DOI:** 10.3389/fcimb.2023.1196905

**Published:** 2023-04-21

**Authors:** Ondrej Cerny, Camilla Godlee, Damián Lobato-Márquez

**Affiliations:** ^1^ Laboratory of Infection Biology, Institute of Microbiology of the Czech Academy of Sciences, Prague, Czechia; ^2^ Department of Infectious Diseases, School of Immunology and Microbial Sciences, King’s College London, London, United Kingdom; ^3^ Department of Infection Biology, London School of Hygiene and Tropical Medicine, London, United Kingdom

**Keywords:** single cell analysis, bacteria-host interaction, cellular microbiology, pathogens, microbiota, flow cytometry, microscopy

Multicellular organisms are very complex systems. This complexity is further increased by their interaction(s) with microbes. Recent technological advances have allowed us to study the interactions inside these complex systems at the single-cell level. This has revealed remarkable heterogeneity of both host and microbial cells, as well as heterogeneity in their interactions. The heterogeneity of interactions between host cells and isogenic bacteria has a large impact on the outcome of host-bacteria interactions.

Our Research Topic focused on the heterogeneity of bacteria-host interaction and consists of six published articles by 32 co-authors with an acceptance rate of approximately 50%.

The interaction between individual host cells and the local microbiome was studied by Li et al. who examined the influence of saliva on the composition of the oral microbiota. First, the authors compared the composition of oral microbiota in mice with excised submandibular or sublingual glands with control animals. The treatment led to a significant increase in *Lactobacilli* in the operated mice, with a stronger effect noticed following submandibular gland removal. The authors further examined the protein composition of individual glands and identified that submandibular glands produce more immune-related proteins than sublingual glands. Single-cell RNA-seq analysis of cells from individual glands identified specific cell types responsible for the production of proteins identified by the proteomic approach. This study shed some light on the influence of individual host cells on the composition of the oral microbiota.

Gu et al. focused on the influence of a bacterial toxin on individual host cells. The authors used flow cytometry and microscopy techniques to study the influence of *Campylobacter jejuni* Cytolethal Distending Toxin (CDT) on the induction of apoptosis in human colon cells. The authors showed high variability in host DNA damage between individual host cells exposed to CDT-containing bacterial lysate. After confirming the cytotoxicity of CDT in their experimental model, the authors identified the Caspase-9/caspase-3/gasdermin E as the executive axis in CDT-induced pyroptosis. Finally, the authors indicated the role of reactive oxygen species in the CDT mechanism of action.

Transmission electron microscopy (TEM) has been used before to study bacteria in great detail. Schulte et al. used TEM in combination with other microscopy techniques to examine morphotypes of individual viable pathogenic bacteria *Salmonella enterica* serovar Typhimurium. Using sample post-contrasting with uranyl acetate and lead citrate, the authors described two morphotypes of bacteria differing in cytoplasmic protein density, which they argued was caused by oxidative stress. Both morphotypes of bacteria were also found inside host cells and showed marks of proliferation. Similarly, the same proportion of both morphotypes was found to be metabolically active. Eventually, a third morphotype was discovered with a dense layer surrounding loose materials in the center. The authors correlated the appearance of bacteria with this “halo-shaped” morphotype with critical environmental conditions in which “the DNA needs to be shielded and protected”. Thus, using single-cell approaches, Schulte et al. provide experimental evidence of a heterogeneous morphological response of individual bacteria to environmental conditions.

Luk et al. reviewed the differences and similarities of *Salmonella* intracellular lifestyles in epithelial cells, fibroblasts, and macrophages. The authors also concentrated on differences between particular cell lines used as models for each category of cells permissive for *Salmonella* intracellular survival. Special interest was given to the escape of *Salmonella* from *Salmonella*-containing vacuoles, the detection of escaped bacteria and their targeting for autophagy, and the hyper-replication of bacteria in the cytosol of epithelial cells. In contrast, cytosolic *Salmonella* does not hyper-proliferate in the cytosol of fibroblasts or immune cells. In addition, the authors described how the activation of caspase pathways by cytosolic *Salmonella*, which leads to host cell death, also differs between epithelial cells, fibroblasts, and immune cells. Another important topic discussed by the authors was the induction of persisters inside different types of host cells.

Persisters were reviewed in more detail by Personnic et al. who chose *Staphylococcus aureus*, *Salmonella enterica*, *Mycobacterium tuberculosis*, and *Legionella pneumophila* as model organisms to describe the mechanisms that induce the formation of persisters in the host. The authors further described how persisters modulate their metabolic and defensive processes to be able to survive prolonged host attacks and how the persisters fight back to inhibit host bactericidal activities. The illustration of different bacterial persister states and host cell functional states underscore the complexity of the covered topic.

Fernández-Fernández et al. reviewed the use of state-of-the-art cell sorting approaches to study *Salmonella* heterogeneity. The authors first introduced the concept of cell sorting and the different types of cell separation methods, including immunomagnetic cell sorting (MACS) and fluorescence-activated cell sorting (FACS). Then, the authors described how these cell separation methods can be applied to investigate bacterial heterogeneity during different types of bacteria-host interactions. The authors also described how cell separation methods can be applied to study the exposure to biocides, the purification of bacterial cell envelopes, and the study of bacterial susceptibility to antibiotics and gene expression. From this review article, it becomes clear that the use of cell sorting has lots of potential to explore key aspects of bacterial physiology that classic microbiology methods (that work at the whole population level) cannot address.

In summary, the articles on this Research Topic brought together interesting observations on interactions between individual host cells and individual bacteria and summarized current knowledge on current technological development as well as on the heterogeneity of bacterial lifestyles upon interaction with the host.

## Author contributions

OC and DL-M conceived and wrote the editorial. CG reviewed and approved the final version.

